# Metformin alleviates nickel-induced autophagy and apoptosis via inhibition of hexokinase-2, activating lipocalin-2, in human bronchial epithelial cells

**DOI:** 10.18632/oncotarget.22317

**Published:** 2017-11-06

**Authors:** Yu-Ting Kang, Wen-Cheng Hsu, Chih-Hsien Wu, I-Lun Hsin, Pei-Ru Wu, Kun-Tu Yeh, Jiunn-Liang Ko

**Affiliations:** ^1^ Institute of Medicine, Chung-Shan Medical University, Taichung, Taiwan; ^2^ Department of Endocrinology and Metabolism, Tungs’ Taichung MetroHarbor Hospital, Taichung, Taiwan; ^3^ Basic Medical Education Center, Central Taiwan University of Science and Technology, Taichung, Taiwan; ^4^ Inflammation Research & Drug Development Center, Changhua Christian Hospital, Changhua, Taiwan; ^5^ Department of Surgical Pathology, Changhua Christian Hospital, Changhua, Taiwan; ^6^ Department of Pathology, Cheng Ching Hospital, Taichung, Taiwan; ^7^ School of Medicine, Chung Shan Medical University, Taichung, Taiwan; ^8^ Department of Medical Oncology and Chest Medicine, Chung Shan Medical University Hospital, Taichung, Taiwan

**Keywords:** nickel, autophagy, hexokinase 2, lipocalin 2, metformin

## Abstract

Autophagy is an intracellular recycling and degradation process for regulating tumor progression, survival and drug resistance. Nickel compounds have been identified as human carcinogens. However, the role of nickel-induced autophagy in lung carcinogenesis has not yet been fully elucidated. In this study, we determined that hexokinase 2 (HK2), which phosphorylates glucose and regulates autophagy, is the key mediator in nickel-induced autophagy in lung bronchial epithelial cells. We attempted to investigate the effects of the antidiabetic drug metformin on HK2 expression and lung cancer chemoprevention. Our results showed that metformin decreases nickel-induced autophagy and activation of apoptosis through inhibition of HK2 gene, protein and activity. Furthermore, we demonstrated that lipocalin 2 (LCN2), which is released by neutrophils at sites of infection and inflammation is involved in HK2-driven autophagy pathway. Knockdown of endogenous HK2 and LCN2 by shRNA reduced nickel-elicited autophagy and apoptosis, illustrating that metabolic alteration and inflammatory action are important in nickel-elicited carcinogenesis. We also determined the association between nickel-induced autophagy and apoptosis. Inhibition of nickel-induced autophagy abolished apoptotic cell death in chloroquine-treated, shLC3 Beas-2B cells and Atg5^−/−^ MFFs. From TGCA database and immunohistochemistry analysis, HK2 and LCN2 expression increased in lung squamous cell carcinoma and their related adjacent normal tissues. Taken together, our results demonstrated that metformin alleviates NiCl_2_-induced autophagy and apoptosis via HK2-driven LCN2 activation in human bronchial epithelial cells. This novel mechanism provides a strategy for targeting nickel-elicited lung cancer progression, as well as for preventing HK2 cumulative damage triggered by environmental carcinogens.

## INTRODUCTION

Autophagy is a highly conserved self-degradative process that packages dysfunctional proteins and organelles into cytoplasmic double-membrane vesicles called autophagosomes. It is activated in response to multiple stimuli in cancer progression, providing a source of nutrients and energy for tumor development during starvation, hypoxia and immune response [1, 2]. Nickel is widely distributed, posing occupational and environmental exposure risks. Nickel (II) compounds have been classified as group I human carcinogens by the International Agency Research in Cancer (IARC) of the World Health Organization (WHO). Human exposure to nickel comes from many sources, such as metal industries, rechargeable batteries, electroplating processes and cigarette smoking [3]. Epidemiological studies have indicated that long-term exposure to nickel compounds is the main reason for raised lung and sinus cancer risks among nickel refinery employees [4, 5]. The molecular carcinogenic mechanisms of nickel toxicity are thought to involve oxidative stress, DNA damage, epigenetic alteration, chronic inflammation and regulation of gene expression [6, 7]. In our previous study, exposure to soluble nickel compound nickel chloride (NiCl_2_) induced epithelial-mesenchymal transition (EMT) in lung epithelial BEAS-2B cells by reactive oxygen species (ROS) generation [8], exhibiting the carcinogenic potential of NiCl_2_. Although the mechanisms of nickel-induced carcinogenesis have been discussed in detail, nickel-activated autophagy has yet to be fully elucidated.

Metabolic alteration is frequently accompanied by rapid differentiation of cells and malignant cells to provide energy. There is a propensity to metabolize glucose to lactic acid by aerobic glycolysis even under sufficient oxygen, a phenomenon known as the Warburg effect [9]. The critical regulator in this frequent cancer phenotype is mitochondrial-bound hexokinase (HK). HK catalyzes the first step of glycolysis, responsible for the conversion of glucose into glucose-6-phosphate. It is well known that HK2, which is found in insulin-sensitive tissues, such as skeletal muscle and adipose tissue, is the major bound HK isoform expressed in cancers [10, 11]. High level of HK2 expression is associated with poor overall survival and prognosis in many types of cancers [12, 13]. Previous studies have indicated that HK2 facilitates autophagy in response to glucose deprivation, functioning as a molecular switch from glycolysis to autophagy under glucose starvation [14]. However, the manner in which nickel-elicited HK2 contributes to lung carcinogenesis by activating autophagy has yet to be thoroughly investigated.

Lipocalin 2 (LCN2) is a critical inflammatory mediator that is persistently induced during endotoxemia, reflecting the extent of kidney damage and kidney failure [15]. LCN2 is also associated with the pathogenesis of various diseases and is upregulated in many types of cancers. Moreover, it has recently been implicated in multiple cancer tumorigeneses [16, 17]. LCN2 also enhance excessive cell autophagy during ischemia/reperfusion injury [18]. LCN2 deficiency decreases autophagy and inhibits cell proliferation [19]. LCN2 may be an important regulator of tumorigenesis through autophagy and proliferation. In the present study, we investigate the effects of exposure to nickel compounds on LCN2-mediated autophagy.

Metformin is the first-line prescribed drug of choice in the treatment of type 2 diabetes and metabolic syndrome, and has recently emerged as a potential anticancer agent with unanticipated cancer prevention activity. Several epidemiological and clinical studies have found that patients using metformin have decreased cancer incidences, as well as inhibited cancer survival and proliferation, in comparison with those using other antidiabetic medications [20–22]. Numerous publications show that anti-carcinogenic effects of metformin are raised by chemical carcinogens or ionizing irradiation in animal models [23, 24].

Although it has been established that nickel exposure upregulates HK2 expression, the mechanism of signal integration between nickel-induced HK2 and autophagy in lung tumor progression has not been elucidated. It exhibits anticancer effect of metformin through regulation of glucose metabolism. Consequently, the aim of this study is to investigate the role of metformin in diminishing NiCl_2_-induced HK2, and associated autophagy and cytotoxicity, in lung bronchial cells. This is the first report of HK2-driven inflammatory cytokine LCN2 expression in the promotion of autophagy under nickel exposure. We also clarify the relationship between NiCl_2_-elicted autophagy and apoptosis, as well as demonstrate the efficiency of metformin in prevention and therapy, following environmental carcinogen exposure.

## RESULTS

### Nickel induces autophagy via HK2 and LCN2 induction in lung cells

**Figure 1:**
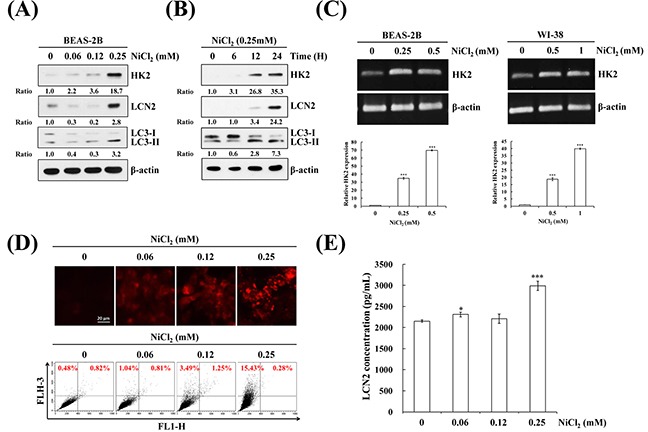
NiCl_2_ induces autophagy and up-regulates HK2 and LCN2 in human bronchial epithelial cells BEAS-2B cells (1×10^6^ cells/ 6 cm dish) were treated with NiCl_2_ in **(A)** dose- and **(B)** time-dependent manner. The protein levels of LCN2, HK2 and LC3B-II were measured on western blot. β-actin was used as an internal control. The relative ratios of LCN2/ β-actin, HK2/ β-actin and LC3B-II/ LC3-I are shown. **(C)** BEAS-2B (1×10^6^ cells/ 6 cm dish) and WI-38 (5×10^5^ cells/ 6 cm dish) were treated with various doses of NiCl_2_ for 48 h. The mRNA level of HK2 was measured on RT-PCR and real-time PCR. Quantification of HK2 by qPCR was normalized to the level of GAPDH, with the average of three independent readings. **(D)** Acridine orange (1μg/mL) was used to stain AVOs in NiCl_2_ (0, 0.06, 0.12, 0.25 mM)-treated BEAS-2B cells (2×10^5^ cells/ 12-well plate) for 48 h. The fluorescence-activated cells were visualized under a red filter fluorescence microscope and quantified by flow cytometry. Scale bar, 20 μm. **(E)** NiCl_2_ increased the secretion of LCN2 in BEAS-2B cell culture supernatant as revealed on ELISA. The data are expressed as mean±SD. ^*^p<0.05, ^***^p<0.001 on two-tailed t test compared with control.

To assess the effects of nickel on cell fate, we evaluated autophagy induction and the generation of relevant proteins in the presence of nickel. Autophagy plays an essential role in lung oncogenesis. At the beginning of autophagy, the cytosolic form of LC3B (LC3B-I, 18 kDa) is converted to the phagophore and autophagosome bound form of LC3B (LC3B-II, 16 kDa). Treatment with various concentrations of nickel for various time periods resulted in cell autophagy in BEAS-2B cells. On western blot, 0.25 mM concentration of nickel significantly induced LC3B-II/LC3B-I ratio after 48 h in BEAS-2B cells (Figure 1A and 1B). In a previous study, nickel accumulation increased cellular glycolytic activity, which is the foremost alteration of energy metabolism in tumorigenesis (the Warburg effect) [25, 26]. In the present study, NiCl_2_ affected multiple genes in the glycolysis pathway on Agilent SurePrint G3 Human V2 GE 8×60K microarray analysis of KEGG pathway. In particular, treatment with NiCl_2_ induced a 38-fold increase in HK2 mRNA level. (Supplementary Materials, Supplementary Table 1 and Supplementary Figure 1). Nickel stimulated HK2 and inflammation protein LCN2 expressions in BEAS-2B cells, as demonstrated on western blot (Figure 1A and 1B). To assess the mRNA levels of HK2 in NiCl_2_-treated cells, BEAS-2B cells and WI-38 fibroblasts were treated with various concentrations of NiCl_2_ for 48 h and analyzed on RT-PCR and Q-PCR (Figure 1C). To confirm autophagic flux in NiCl_2_-treated cells, AVO development was detected by staining of late autophagic vacuoles with acridine orange dye. As shown in Figure 1D upper, nickel prompted AVO formation in BEAS-2B cells. To calculate the AVO fractional volume after NiCl_2_ treatment, flow cytometric analysis was performed. The data indicated that NiCl_2_ stimulates AVO development in a dose-dependent manner in BEAS-2B cells (Figure 1D lower). Secreted LCN2 levels increased following 0.25 mM NiCl_2_ treatment in BEAS-2B cells (Figure 1E). These results implied that HK2 and LCN2 are activated in the presence of NiCl_2_.

### Metformin represses autophagy and apoptosis by inhibition of NiCl_2_-induced HK2 levels and activity

**Figure 2:**
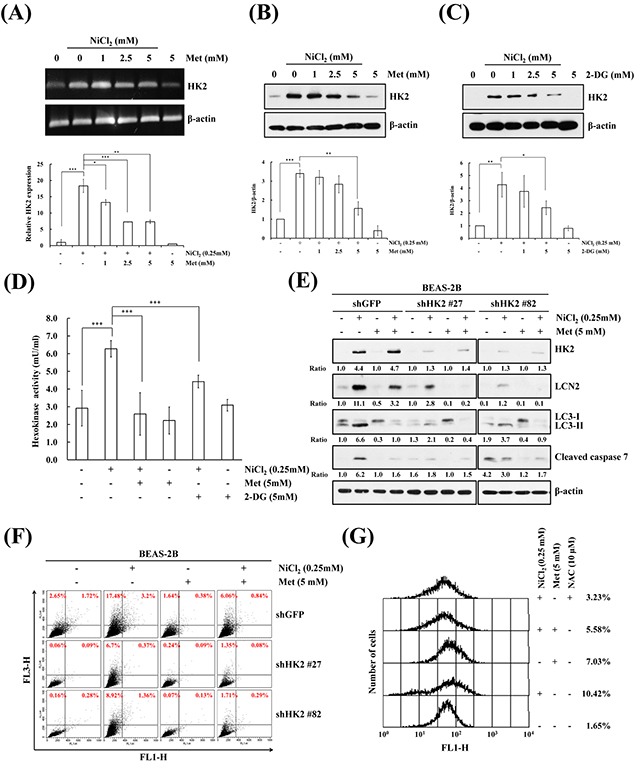
Inhibition of NiCl_2_-induced hexokinase 2 represses autophagy and apoptosis **(A)** BEAS-2B cells (1×10^6^ cells/6 cm dish) were treated with NiCl_2_ (0, 0.25 mM) and metformin (0, 1, 2.5, 5 mM) for 48 h. The mRNA levels were measured on RT-PCR and real-time PCR. ^*^p<0.05, ^**^p<0.01, ^***^p<0.001, two-tailed t test. **(B, C)** BEAS-2B cells (1×10^6^ cells/6 cm dish) were co-treated with 0.25 mM NiCl_2_, metformin (0, 1, 2.5, 5 mM) or 2-DG (0, 1, 2.5, 5 mM) for 48 h. The protein levels were determined on western blot analysis. β-actin was used as the internal control. Statistical analysis of western blotting. The protein levels of HK2 were standardized by β-actin protein level. ^*^p<0.05, ^**^p<0.01, ^***^p<0.001, two-tailed t test. **(D)** Quantification of HK activity from whole-cell lysates showed a significant decrease following treatment with 0.25 mM NiCl_2_ combined with 5 mM metformin or 5 mM 2-DG for 48 h. The level of HK activity was determined by OD 450. ^***^p<0.001, two-tailed t test. **(E)** After 0.25 mM NiCl_2_ and 5 mM metformin treatment, equal amounts of total lysates from BEAS-2B shGFP and shHK2 cells (1×10^6^ cells/6 cm dish) were analyzed on western blot. The relative ratios of HK2/β-actin, LC3-II/LC3-I and cleaved caspase 7/β-actin are shown. **(F)** Flow cytometric analysis of the NiCl_2_- and metformin-treated cells after staining with acridine orange for the quantification of AVOs. **(G)** BEAS-2B cells were pretreated with 10 mM NAC for 1 h followed by exposure to 0.25 mM NiCl_2_ and 5 mM metformin for 48 h. The intracellular ROS levels of the cells were measured by flow cytometry analysis staining with H_2_DCFDA.

HK catalyzes the first step of glycolysis, phosphorylating glucose to glucose-6-phosphate. HK2 plays an important role not only in glycolysis, but also in cell survival. We investigated whether HK2 is involved in NiCl_2_-induced cell fate. NiCl_2_ increased HK2 mRNA levels and metformin diminished NiCl_2_-induced HK2 expression in a dose dependent manner (Figure 2A upper). The same results were obtained on real time RT-PCR for the detection of mRNA expression of HK2 in BEAS-2B cells (Figure 2A lower). As shown in Figure 2B, we performed western blotting to examine whether metformin diminishes up-regulation of the protein levels of HK2 in the presence of NiCl_2_. To quantify the HK2 protein expression affected by NiCl_2_ and metformin on western blotting, we repeated the same experiment three times. As 2-DG is an analog of glucose, it has been used as an HK inhibitor, as it competes with glucose. Remarkably, 2-DG treatment also decreased NiCl_2_-induced HK2 expression (Figure 2C). As shown in Figure 2D, metformin and 2-DG significantly decreased NiCl_2_-induced HK activity. Moreover, the decrease in HK2 expression was based on two different short hairpin RNAs (shRNAs). The responses of BEAS-2B shGFP cells were similar to those of parental BEAS-2B cells after NiCl_2_ and metformin treatment. In BEAS-2B shHK2 cells, NiCl_2_-elicited LC3B-II and cleaved caspase-7 expressions were significantly diminished. In addition, there was strong correlation between the expression of HK2 and LCN2 level. Moreover, BEAS-2B shHK2 cells significantly decreased following NiCl_2_ treatment (Figure 2E). To confirm the effect of HK2 silencing on AVO fraction volume after NiCl_2_ and metformin treatment, flow cytometry was performed (20.6% *versus* 7.1% and 10.4%) (Figure 2F). These data indicated that HK2 is involved in the induction of autophagy in the presence of NiCl_2_. It is well known that the generation of reactive oxygen species (ROS) contributes to nickel-triggered carcinogenesis, including EMT promotion and the cause of DNA damage [8, 27]. To determine whether metformin can suppress NiCl_2_-induced ROS accumulation, cells were treated with 2′, 7′ -dichlorodihydrofluorescein diacetate (H_2_DCFDA) and analyzed by flow cytometry. Results revealed that metformin decrease ROS generation in the presence of nickel (10.42% *versus* 5.58%). N-acetyl-cysteine (NAC, 1 mM), the ROS scavenger, was used to confirm the reversion of NiCl_2_-induced ROS (Figure 2G).

### Endogenous LCN2, but not exogenous LCN2, triggers NiCl_2_-mediated autophagy in bronchial epithelial cells

**Figure 3:**
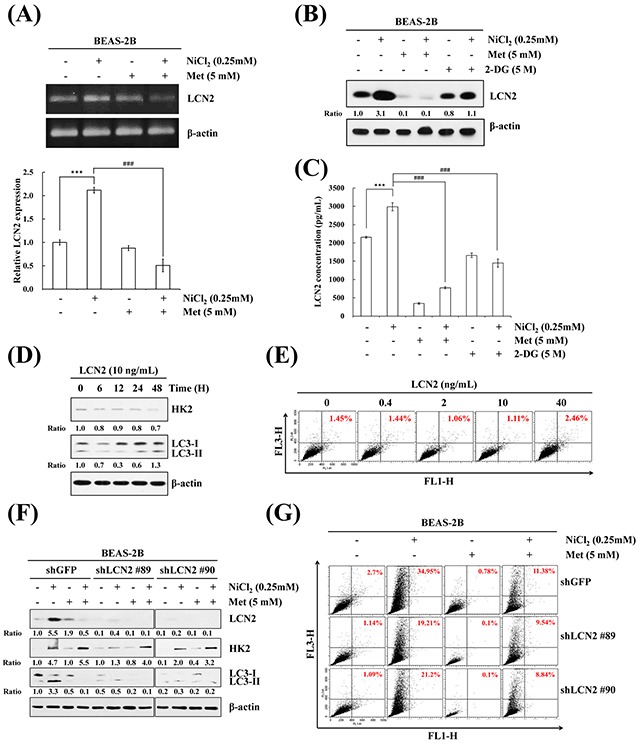
LCN2 is involved in NiCl_2_-induced autophagy in human bronchial epithelial cells **(A)** BEAS-2B cells (1×10^6^ cells/ 6 cm dish) were treated with NiCl_2_ (0, 0.25 mM) and metformin (0, 5 mM) for 48 h. The mRNA level of LCN2 was determined on RT-PCR and qPCR. Quantification of LCN2 by qPCR was normalized to the level of GAPDH, with the average of three independent readings. The data are expressed as mean ± S.D. ^*^p<0.05, ^***^p<0.001 on two-tailed t test. **(B)** BEAS-2B cells (1×10^6^ cells/ 6 cm dish) were treated with NiCl_2_ (0, 0.25 mM) and metformin (0, 5 mM) or 2-DG (0, 5 mM) for 48 h and subjected to western blotting for LCN2. **(C)** Metformin or 2-DG decreased NiCl_2_-induced secretion of LCN2 in BEAS-2B (2×10^5^ cells/12-well plate) cell culture supernatants, as revealed on ELISA. The data shown are the mean ± S.D. of three independent experiments. ^***^p<0.001 compared to control and ^###^p<0.001 compared to Ni-treated cells, two-tailed t test. **(D)** Cells (1×10^6^ cells/6 cm dish) were treated with LCN2 (10 ng/mL) for 0, 6, 12, 24, 48 h. HK2, LC3B levels were analyzed on western blot. β-actin served as the loading control. **(E)** BEAS-2B cells (1×10^6^ cells/ 6 cm dish) were treated with 0, 0.4, 2, 10, 40 ng/mL LCN2 for 48 h. The cells were stained with acridine orange (1 μg/mL) for AVO observation and analyzed using flow cytometry. **(F)** BEAS-2B cells (1×10^6^ cells/ 6 cm dish) were treated with NiCl_2_ and metformin for 48 h after infection with lentivirus carrying shLCN2 #89, shLCN2 #90 or vector control (shGFP). The protein levels of LCN2, HK2, LC3B were detected on western blot. β-actin was used as an internal control. **(G)** Quantitative detection of AVOs by acridine orange staining in cells was performed using flow cytometry.

LCN2, also known as neutrophil gelatinase-associated lipocalin (NGAL), is required for tumor progression and metastasis. It is often implicated in the responses to hypoxia and apoptosis induction [28]. However, the correlation between LCN2 and autophagy in the presence of NiCl_2_ remains unclear. Actually, a causal link between LCN2 and HK2 levels and autophagy levels in bronchial epithelial cells has not been reported, which prompted us to clarify whether LCN2 is involved in NiCl_2_-elicited autophagy. To assess the effect of metformin on NiCl_2_-induced LCN2 expression, BEAS-2B cells were cultured in the presence of NiCl_2_ with or without metformin for 48 h, and RT-PCR and qPCR were performed to detect the mRNA expressions of LCN2. As shown in Figure 3A, metformin significantly decreased NiCl_2_-induced LCN2. In addition, treatment with metformin or 2-DG reduced NiCl_2_-elicited LCN2 protein levels. The same results were obtained on ELISA for secretion of LCN2 in a BEAS-2B cell culture supernatant following NiCl_2_, metformin or 2-DG treatment (Figure 3B, 3C). Particularly, the protein level of LCN2 was down-regulated approximately 40% in all cell lysates following 2-DG treatment. However, in metformin-treated cells, NiCl_2_–induced secretion of LCN2 was blocked (Figure 3B, 3C). These results revealed that NiCl_2_–mediated LCN2 is repressed by metformin at translational and transcriptional levels.

We further examined whether LCN2 independently prompts autophagy and attempted to clarify the relationship between LCN2 and HK2 in lung epithelial cells. Western blot analysis was performed to observe whether LCN2 treatment induces the processing of LC3B-I to LC3B-II, and the protein expression of HK2. The results demonstrated that HK2 and LC3B-II/I do not accumulate following LCN2 treatment (10 ng/mL) for up to 48 h (Figure 3D). We utilized acridine orange staining to confirm this result. Concentrations of LCN2, up to 40 ng/mL, failed to alter the increase in AVO accumulation (Figure 3E). Furthermore, the protein levels of HK2 and LC3B-II/I ratios were determined after attenuation of LCN2 activity by small hairpin RNA (shRNA). The data revealed that blockade of endogenous LCN2, but not exogenous LCN2, represses autophagy in BEAS-2B cells (Figure 3F). To calculate the AVO fractional volume, we performed flow cytometric analysis 48 h after LCN2 silencing and co-treatment with NiCl_2_ and metformin (Figure 3G). Taken together, the results suggested that LCN2 is involved in NiCl_2_-induced autophagy.

### NiCl_2_-mediated autophagy decreases following metformin treatment in human bronchial epithelial cells

**Figure 4:**
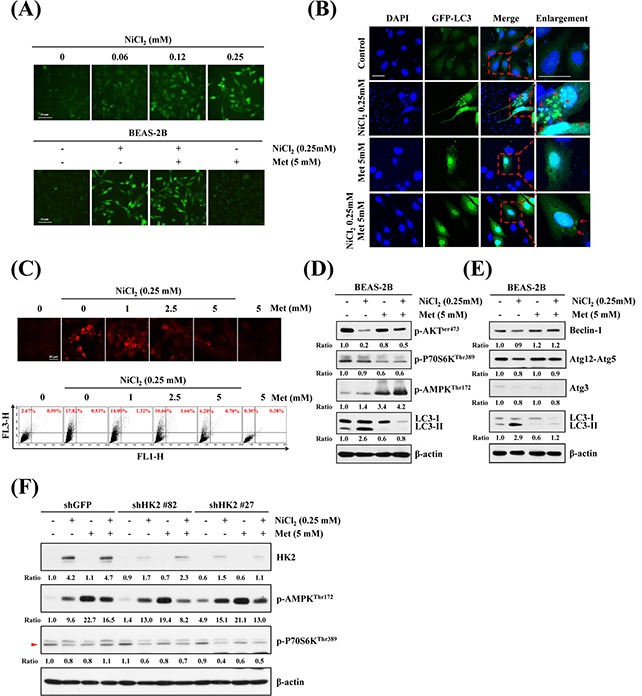
Effects of metformin on NiCl_2_-mediated autophagy in human bronchial epithelial cells **(A)** BEAS-2B cells (2×10^5^ cells/well of 12-well plate) were treated with varying doses of NiCl_2_ (0, 0.06, 0.125, 0.25 mM) (top), or with NiCl_2_ (0, 0.25 mM) and metformin (0, 1, 2.5, 5 mM), for 48 h (bottom). The cells were stained with Newport Green™ DCF diacetate (1 μM) for green fluorescence observation under a green filter fluorescence microscope. Scale bar, 20 μm. **(B)** pEGFP-LC3B transfection revealed LC3B puncta (green) in BEAS-2B cells (8×10^4^ cells/well of 24-well plate) treated with NiCl_2_ (0, 0.25 mM) and metformin (0, 1, 2.5, 5 mM) for 48 h. Cells were fixed and stained with DAPI for nuclear visualization under a confocal microscope. Dashed lines encircle the enlarge images and arrow heads point to the puncta of GFP-LC3. Scale bars, 20 μm. **(C)** BEAS-2B cells (1×10^6^ cells/ 6 cm dish) were treated with NiCl_2_ (0, 0.25 mM) and metformin (0, 1, 2.5, 5 mM) for 48 h and stained with acridine orange (1 μg/mL) for AVO observation. Cells were visualized under a red filter fluorescence microscope with quantification by flow cytometry. **(D)** Western blots of p-AKT, p-P70S6K, p-AMPK, LC3B and **(E)** autophagy-related genes Beclin-1, Atg12-Atg5 and Atg3 expressions in protein lysates from BEAS-2B cells (1×10^6^ cells/ 6 cm dish) treated with NiCl_2_ (0, 0.25 mM) and metformin (0, 5 mM) for 48 h. β-actin was used as an internal control. The relative ratios of p-AKT/β-actin, p-P70S6K/β-actin, p-AMPK/β-actin, LC3B-II/β-actin, Beclin-1/β-actin, Atg12-Atg5/β-actin and Atg3/β-actin are shown. **(F)** p-AMPK and p-P70S6K were determined by western blotting after BEAS-2B shGFP and shHK2 cells (1×10^6^ cells/ 6 cm dish) were treated with NiCl_2_ and metformin for 48 h. β-actin was used as an internal control.

To determine the intracellular distribution of ionic nickel in the cells, we utilized Ni^2+^-selective fluorescence dye Newport Green^TM^ DCF, which fluoresces when there is binding with ionic nickel ions. As shown in Figure 4A upper, in a dose-dependent experiment, BEAS-2B cells were exposed to various concentrations of NiCl_2_ for 48 h. The result was an incremental increase in green fluorescence in NiCl_2_-treated cells. In a previous study, cellular responses of metformin were associated with this drug's metal-binding properties, especially binding with copper. However, the association with nickel remained unclear [29]. To further investigate whether metformin sequesters intracellular nickel, BEAS-2B cells were co-treated with 0.25 mM NiCl_2_ and 5 mM metformin for 48 h, then stained with Newport Green^TM^ DCF. As shown in Figure 4A lower, treatment with metformin did not affect NiCl_2_-activated fluorescence, indicating that metformin does not contribute to blockage of nickel ions into cells. To evaluate the effect of metformin on NiCl_2_-elicited autophagy, we utilized pEGFP-LC3 transient transfection to visualize aggregation of expression of LC3B. After treatment with 0.25 mM NiCl_2_, GFP-LC3 was redistributed from a ubiquitous, diffuse pattern toward autophagosomes, observed as cytoplasmic dots in BEAS-2B cells. Remarkably, treatment with 5 mM metformin decreased the formation of GFP-LC3 puncta (Figure 4B). We then examined the effect of metformin on NiCl_2_-induced AVO development. Treatment with metformin blunted AVO formation in a dose-dependent manner in NiCl_2_-treated cells. Flow cytometric analysis was performed to quantify the AVO fractional volume (18.35% *versus* 6.98%) (Figure 4C).

mTOR, the Akt and AMPK downstream effector, plays a critical role in cell proliferation, growth and survival. Activated mTOR promotes protein translation by phosphorylating its substrates, including p70 ribosomal protein S6 kinase (p70S6K) [30]. To identify the effects of metformin on NiCl_2_-induced autophagy-related pathway and genes, NiCl_2_ and metformin were administered for 48 h, followed by analysis of protein expressions on western blotting. As shown in Figure 4D and Figure 4E, Akt-Ser473 phosphorylated level was downregulated, but did not affect phosphorylated p70S6K-Thr389, AMPK-Thr172, beclin-1, ATG12-ATG5 and ATG3 levels after 0.25 mM NiCl_2_ treatment for 48 h. Beclin-1, ATG12-ATG5 and ATG3 are members of the ATG family involved in the formation of autophagosomes [31]. Metformin treatment revived the Akt-Ser473 phosphorylated expression, but reduced NiCl_2_-mediated autophagy, perhaps through Akt-dependent but mTOR-independent pathway. In addition, metformin is known to activate AMPK. To determine the relationship between HK2 and p-AMPK on nickel-induced autophagy, shGFP and shHK2 BEAS-2B cells treated with NiCl_2_ and metformin were performed. As shown in Figure 4F, there were no significant changes in the protein levels of p-AMPK and p-P70S6K after knockdown of HK2.

### NiCl_2_-elicted autophagy contributes to activation of apoptosis

**Figure 5:**
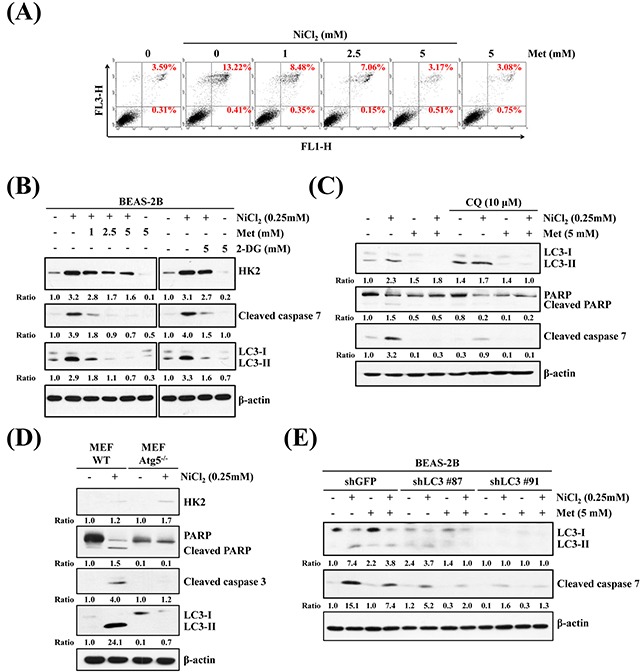
Inhibition of autophagy decreases NiCl_2_-elicted apoptosis **(A)** Flow cytometry analysis of BEAS-2B cells (1×10^6^ cells/ 6 cm dish) treated with NiCl_2_ (0, 0.25 mM) and metformin (0, 5 mM) for 48 h, after which cells were stained with Annexin V/PI. **(B)** BEAS-2B cells (1×10^6^ cells/ 6 cm dish) were treated with NiCl_2_ (0, 0.25 mM) and metformin (0, 1, 2.5, 5 mM) or 2-DG (0, 5 mM) for 48 h and subjected to western blotting for HK2, cleaved caspase 7 and LC3B. β-actin was used as an internal control. The relative ratios of HK2/β-actin, cleaved caspase 7/β-actin and LC3B-II/LC3-I are shown. **(C)** Western blot of BEAS-2B cells (1×10^6^ cells/6 cm dish) treated with NiCl_2_ (0, 0.25 mM) and metformin (0, 5 mM) with or without CQ (0, 10 μM) for 48 h. **(D)** Atg5 wild type (WT) and Atg5^−/−^ MEF cells (2×10^5^ cells/6 cm dish) were treated with NiCl_2_ (0, 0.25 mM) for 48 h. β-actin was used as an internal control. The relative ratios of HK2/β-actin, LC3B-II/LC3B-I, cleaved PARP/β-actin and cleaved caspase 3/β-actin are shown. **(E)** Cleaved caspase 7 and conversions of LC3-I to LC3-II were determined by western blotting after BEAS-2BshLuc and shLC3 cells (1×10^6^ cells/ 6 cm dish) were treated with NiCl_2_ and metformin for 48 h. β-actin was used as an internal control.

It is well documented that nickel exposure induces apoptosis via ROS accumulation and involvement of mitochondria, ER-stress, Fas, and c-Myc [32]. To investigate the effects of metformin on NiCl_2_-mediated apoptosis and to clarify the cellular sources of HK2 in autophagy and apoptosis in NiCl_2_-treated cells, BEAS-2B cells were treated with 1, 2.5 or 5 mM metformin, with or without 0.25 mM NiCl_2_, for 48 h and analyzed by flow cytometry. As shown in Figure 5A, NiCl_2_-mediated apoptosis was recovered by metformin (13.22% *versus* 3.17%). In addition, we assessed the protein markers of apoptosis and autophagy in the presence of NiCl_2_, with or without metformin or 2-DG, on western blotting. Metformin or 2-DG treatment simultaneously abolished the increases in LC3-I/LC3-II ratio and cleaved caspase-7 expression in NiCl_2_-treated BEAS-2B cells (Figure 5B). To further determine whether NiCl_2_-mediated autophagy stimulates apoptosis, the autophagy inhibitor chloroquine (CQ) was used to suppress late phase autophagy. As shown in Figure 5C, treatment with NiCl_2_ and metformin, as well as with 10 μm CQ, blocked the endogenous LC3-II turnover and resulted in increased NiCl_2_-elicted autophagy. CQ treatment also inhibited NiCl_2_-mediated cleavage of poly ADP-ribose polymerase and cleavage of caspase-7, which served as apoptotic markers. Particularly, metformin prevented the accumulation of LC3-II and apoptotic proteins, with or without CQ treatment. To further confirm the correlation between autophagy and apoptosis in cells exposed to NiCl_2_, we observed atg5 knockout mouse embryonic fibroblast cells (Atg5^−/−^ MEF cells), as well as LC3 knocked-down BEAS-2B cells. In atg5^−/−^ and atg5 wild-type MEF cells, NiCl_2_ treatment slightly increased the protein expression of HK2. Similar to NiCl_2_-treated BEAS-2B cells, in atg5 WT MEFs there was significant induction of LC3 I to II conversion, as well as cleavage of PARP and caspase 3 expression. However, there was failure to prompt atg5^−/−^ MEFs (Figure 5D). As shown in Figure 5E, the specific shRNA targeting LC3 was transfected into BEAS-2B cells with knockdown of the expression of LC3. In comparison with BEAS-2B shGFP cells, NiCl_2_-induced cleavage of caspase 7 was blunted in BEAS-2B shLC3 cells. Our results demonstrated that NiCl_2_-induced autophagy induces apoptosis.

### HK2 is the crucial regulator in lung cancer progression

**Figure 6:**
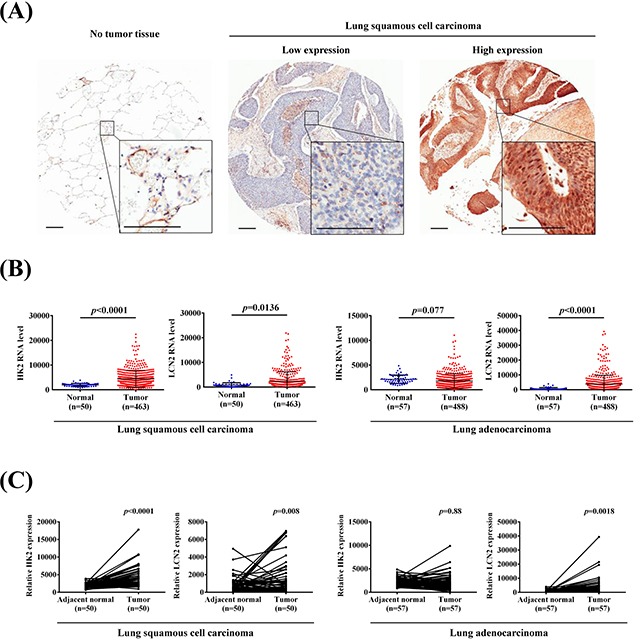
HK2 and LCN2 levels increased in lung squamous cell carcinoma and lung adenocarcinoma samples **(A)** IHC HK2 protein expressions in NSCLC patients. (Left) No tumor tissue. (Middle) Low expression immunostaining. (Right) High expression immunostaining. Scale bars, 200 μm. **(B)** The expressions of HK2 and LCN2 in normal and lung squamous cell carcinoma and adenocarcinoma from TCGA Data Portal. **(C)** Relative expressions of HK2 and LCN2 in 50 or 57 pairs of lung squamous cell carcinoma or adenocarcinoma tumor tissues and their corresponding adjacent non-cancerous tissues.

We assessed HK2 and LCN2 expressions in The Cancer Genome Atlas (TCGA) Data Portal from Broad GDAC Firehose and performed immunohistochemical staining to detect the expressions of HK2 and LC3B in 72 human lung cancer specimens to determine whether HK2, LCN2 and LC3B are involved in lung cancer progression. The representative IHC results are shown in Figure 6A. The presence or absence of HK2 and LC3B protein expressions was associated with tumor stage, T status and metastasis (Supplementary Table 2). Both HK2 and LCN2 expressions significantly increased in cancer tissues when compared with normal tissues in lung squamous cell carcinoma (Figure 6B). Furthermore, we examined the expressions of HK2 and LCN2 in LUSC and LUAD tissues and their corresponding noncancerous tissues using the TCGA Data Portal (Figure 6C). The results revealed that HK2 and LCN2 are associated with tumor progression, especially in LUSC tissue.

## DISCUSSION

It is well documented that metformin alleviates autophagy and apoptosis via HK2 and LCN2, following exposure to nickel, in bronchial epithelial cells. The molecular basis of nickel carcinogenicity has proven complex, as many chronic inflammation and stress response pathways are activated in nickel-specific toxicology profiles. There is much evidence that chronic inflammation contributes to the onset and progression of cancer [33, 34]. In recent studies, Toll-like receptor 4 (TLR4) has been identified as the critical mediator of the innate immune response to nickel that triggers NF-κB signaling and pro-inflammatory gene activation [35]. Although nickel compounds have low mutagenic capabilities, previous studies have found that nickel accumulation in lung tissues contributes to incremental levels of EGFR and P53 mutations, which can reduce DNA repair activity and promote tumor invasion, leading to lung carcinogenesis [36–38]. In addition, accumulating evidence has emphasized the importance of nickel in modulating the epigenetic landscape that includes chromatin structural modifications, DNA methylation and histone modifications [39, 40]. Nickel also induces the up-regulation of a specific set of proteins and microRNAs (miRNAs), leading to altered DNA methylation and histone modification landscapes in a variety of cell types [7, 39]. From the results of recent studies, HK2 is highly expressed in various cancers, and is regulated by miRNA [41]. MiR-143, an anti-oncomiR, is often downregulated in cancers, such as colon and gastric cancers, as well as B-cell lymphoma [42–44]. It targets HK2 mRNA and inhibits HK2 expression [41, 45]. Moreover, other miRNAs may be involved in the altered expression of HK2 in tumors, including miR-181b, miR-125b and miR-182 [46–48].

Nickel-induced carcinogenesis may involve glycolysis pathway activation. It has been shown that genes related to glucose metabolism and glycolysis are inducible by nickel exposure in an HIF-dependent manner [49]. Actually, HIF has been found to accumulate in various cell lines in the presence of nickel [50]. Increasing numbers of studies have shown that metabolic enzymes directly contribute to carcinogenesis. In comparison with normal tissues, cancer cells prefer to metabolize glucose into lactic acid by glycolysis, which is known as the “Warburg effect”, and is accompanied by upregulation of HK2 [9]. Previous studies have demonstrated that HK2 is highly present in lung and breast cancers, and is required for tumor initiation and maintenance. Tumor progression is impaired following its downregulation [51]. Here, we demonstrated that reduction in NiCl_2_-induced HK2 by metformin inhibits NiCl_2_-mediated autophagy. It is known that metformin suppresses hypoxia-induced HIF-1α accumulation [52]. In this study, we confirmed that metformin decreases nickel-induced HIF-1α expression (data not shown). We also demonstrated the importance of HK upregulation in nickel-induced autophagy through inhibition of the expression and activity of HK2 using competitive HK inhibitor 2-DG and HK2-specific shRNA silencing. HK2 silencing combined with metformin demonstrated that autophagy is not only inhibited by HK2 activation (Figure 2E).

A previous study demonstrated that HK2 positively regulates protective autophagy via TORC1 inhibition in response to glucose deprivation [14]. However, in contrast with normal tissues, inhibition of HK2 by 2-DG suppresses lung cancer cell growth through induction of cell apoptosis and autophagy [53]. Furthermore, treatment with 2-DG and CQ represses HK2-mediated Warburg effect and ULK1-dependent autophagy activates apoptosis to cause tumor regression [54]. These studies indicated that HK2 is able to regulate different effects of autophagy and is a key mediator and energy precursor. Therefore, we investigated metformin as a new anti-autophagy drug by targeting Ni-accumulated HK2 in lung epithelial cells.

In our previous study, treatment with NiCl_2_ stimulated EMT via HIF-1α-dependent pathway and *E-cadherin* promoter hypermethylation in bronchial epithelial cells [8]. EMT is an important step in the progression of lung cancer toward metastasis and invasion and occurs during the development of epithelial carcinogenesis [55, 56]. Actually, the correlation between EMT and autophagy has been well studied over the past decade. It has been observed that autophagy contributes to cancer invasion through EMT activation during starvation or hypoxia [57–59].

A recent report showed that HK2 overexpression is associated with the hypomethylation status of CpG island region −379 to +209 from HK2 promoter in hepatocellular carcinomas [60]. It had been stated that DNA hypomethylation of proto-oncogene contribute to nickel-induced malignant transformation [61]. We could not rule out the possibility that nickel will regulate the epigenetic alteration of HK2 promoter hypomethylation. In addition, metformin may obstruct HIF-1α binding on HK2 promoter resulting in HK2 decrease. Overall, these results evidence that the potential capacity of nickel alter the epigenetic regulation and the transition to the status of higher aggressiveness and thus progression of carcinogenesis.

It is worth noting that autophagy induced by TLR4 signaling promotes TLR-triggered cytokine production, which accelerates migration and invasion of lung cancer cells [62]. Studies have suggested that LCN2 upregulated by LPS is influenced via TLR4 signaling pathway [63, 64]. In this study, we demonstrated that NiCl_2_ induces autophagy induction via increment levels of HK2 and LCN2. As shown in Figure 2F and Figure 3F, we used acridine orange stain to verify the role of HK2 and LCN2 in nickel-induced autophagy after HK2 and LCN2 gene silencing. The results demonstrated that NiCl_2-_elicited autophagy is via HK2-LCN2 pathway. Although we suggested that LCN2 is involved in NiCl_2_–induced autophagy pathway, we could not clarify whether nickel upregulates LCN2 expression via TLR4 signaling or if nickel-induced autophagy and EMT activation are facilitated by TLR4 at the source. We did find that metformin restores the protein expression of E-cadherin (data not shown). In future studies, we will explore the precise mechanism of metformin in diminishing the various effects of nickel.

Nickel induced malignant transformation through SQSTM1/P62 and inflammatory TNF upregulation in Beas-2B cells [65]. Son et al. found that nuclear factor erythroid 2-related factor 2 (Nrf2) plays an important role in nickel-induced autophagy [66]. In the present study, we found that nickel induce autophagy via HK2 and LCN2. Autophagy is triggered by the stress of metabolism and inflammation. Our results suggest that autophagy is one of cancer-promoting reasons under nickel exposure. In fact, autophagy has been shown to play a protective role against apoptosis in malignant transformation. Whether autophagy promotes cell survival or cell death depends on the levels of stress. When stress severity or duration increases, cell death may result. In brief, autophagy affects cellular homeostasis [67, 68]. From our data, nickel simultaneously induces autophagy and apoptosis. To further demonstrate the association of autophagy with apoptosis in the presence of nickel, endogenous LC3 was knocked down by shRNA treatment, and WT or Atg5^−/−^ MEF cells were treated with CQ (Figure 5C-5E). We observed that the activation time point of nickel-induced autophagy is earlier than that of apoptosis (data not shown). We used short-term exposure and high concentrations of nickel to clarify the role of nickel-induced autophagy. In the presence of excessive nickel, there is an imbalance in autophagy, which promotes apoptosis.

There are some limitations in the study. Nickel contents were hardly determined in tissue array. We could not analyze the samples for the relationship of nickel exposure and HK2 expression. However, we investigate the tumorigenesis of HK2 and LCN2 on TCGA database (Figure 6B, 6C). Both HK2 and LCN2 serve as biomarkers in lung cancer progression. We also observed the expression of HK2, LCN2 and autophagy-related genes in lung cancer cell lines, including CL1-0, CL1-5, TL-6 and H1975 with or without 0.25 mM NiCl_2_ (Supplementary Figure 2A, 2B). Results revealed that most of cancer cells elevate the expression of HK2 and LC3B in the presence of nickel. Equivalently with BEAS-2B cells, autophagy-related genes Atg5 and Beclin-1 except LC3B were decreased after NiCl_2_ treatment.

**Figure 7:**
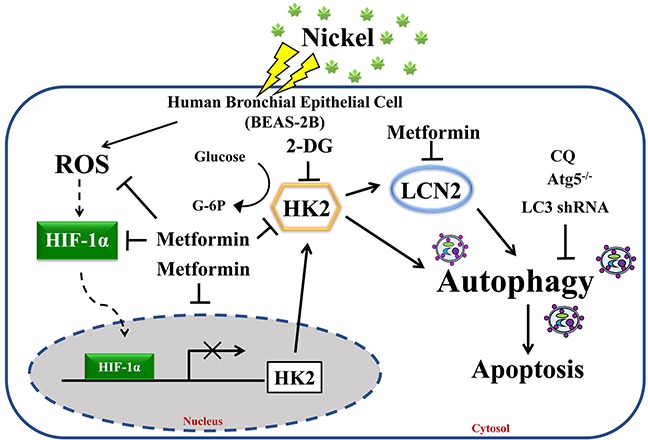
Proposed model and hypothesis of metformin-induced repression of HK2-driven autophagy following nickel exposure in BEAS-2B cells Nickel-induced HK2 is a potent mediator of autophagy activation in BEAS-2B cells. We demonstrated the therapeutic efficacy of metformin in the prevention of toxicity of environmental carcinogens.

In conclusion, the results of this study provide evidence that metformin alleviates NiCl_2_-stimulated autophagy via the inhibition of HK2 and LCN2 expressions (Figure 7). Accumulation of nickel triggers metabolic changes and inflammatory environment contributes to lung cancer development. The results of this study also demonstrated the preventive effects of metformin against cumulative damage caused by environmental carcinogens.

## MATERIALS AND METHODS

### Human lung specimens and cell lines

Human bronchial epithelial cell line BEAS-2B (CRL-9606^TM^, ATCC) and lung fibroblast cell line WI-38 (CCL-75™, BCRC) were purchased from the American Type Culture Collection and BCRC (Taiwan), respectively. Tissue specimens were collected in tissue microarray (SuperBiochips) obtaining from GenDiscovery. Tumor types and stages were determined according to WHO classification (World Health Organization, 1981) by experienced clinical pathologists. This study was approved by the Chung Shan Medical University Hospital Institutional Review Board (CSMUH IRB CS16101).

### Cell culture and chemicals

BEAS-2B cells were routinely grown in serum-free LHC-9 medium (GIBCO, 12680-013). WI-38 cells were cultured in Minimum Essential Medium (MEM) (GIBCO, 11095-080) containing 10% heat-inactivated fetal bovine serum (FBS) (GIBCO, 10437), 100 ng/mL each of penicillin and streptomycin (GIBCO, 15140), 1 mM nonessential amino acid and 1 mM sodium pyruvate. Both types of cells were incubated in humidified atmosphere of 5% CO_2_ at 37^°^C. Reagents used in the study included NiCl_2_ (Sigma, N6136), metformin (Sigma, D150959), 2-deoxy-D-glucose (2-DG, Sigma, D6134), chloroquine (CQ) diphosphate salt (Sigma, C6628), and recombinant human LCN2 (Biolegend, 588102).

### Western blot analysis

Following treatment protocols, cells were washed twice with ice-cold PBS (2.7 mMKCl, 2 mM KH_2_PO_4_, 137 mMNaCl, 10 mM Na_2_HPO_4_, pH 7.4), then lysed with RIPA buffer (2 mM pH 8.0 ETDA, 50 mMTris pH 7.4, 150 mMNaCl, 1% NP-40, 1 mM PMSF, 1% sodium deoxycholate) containing protease inhibitor cocktail (Roche, 04693116001). The concentration of protein extracts was assayed with Bio-Rad Protein Assay Kit (Bio-Rad, 500-0006). Cell lysates were resolved by sodium dodecyl sulfate polyacrylamide gel electrophoresis and transferred to hydrophobic polyvinylidene fluoride transfer membrane (PVDF) (PALL, 29301-808). Membranes were blocked in TBS containing 5% non-fat milk and 0.2% Tween 20 for 1 h and probed overnight at 4^°^C with primary antibodies. Antibodies against HK2 (2867), PARP (9542), LC3B (3868), cleaved caspase-7 (9491), cleaved caspase-3 (9661), p-AKT^Ser473^ (9271) and p-AMPKα^Thr172^ (2535) were obtained from Cell Signaling Technology. Antibody against p-p70S6k^Thr389^ (MABS82) was purchased from Millipore. Antibody against LCN2 (AF1757) was supplied by R&D Systems and β-actin (A5441) was supplied by Sigma. Blots were washed in 0.2% TBS-Tween 20 and incubated with HRP-conjugated secondary antibodies for 1 h. The protein blots were visualized using enhanced chemiluminescence reagent obtained from Perkin Elmer (NEL105001EA).

### RNA extraction, reverse transcription-PCR and quantitative real time-PCR analysis

Total RNA was extracted from harvested cells using the rare RNA reagent (Genepure Technology) per the manufacturer's instructions. We quantified RNA and reverse transcribed cDNAs from 3 μg of total RNA per 20 μL RT reaction using High Capacity cDNA Reverse Transcription Kit (Applied Biosystems, 4368813). PCR was carried out with the following primers: HK2 forward: 5′-ATG AGG GGC GGA TGT GTA TCA-3′, reverse: 5′-GGT TCA GTG AGC CCA TGT CAA-3′; LCN2 forward: 5′-GAG TTA CCC TGG ATT AAC GA-3′, reverse: 5′-CTC CTT TAG TTC CGA AGT CA-3′; LC3B forward: 5′-GCC GTC GGA GAA GAC CTT CAA G-3′, reverse: 5′-TGG TGT GGA GAC GCT GAC CAT G-3′; Atg5 forward: 5′-TTT GCA TCA CCT CTG CTT TC-3′, Atg5 reverse: 5′-TAG GCC AAA GGT TTC AGC TT-3′; Beclin-1 forward: 5′-GGC TGA GAG ACT GGA TCA GG −3′, Beclin-1 reverse: 5′-CTG CGT CTG GGC ATA ACG-3′; β-actin forward: 5′-TCA TCA CCA TTG GCA ATG AG-3′, reverse: 5′-CAC TGT GTT GGC GTA CAG GT-3′. PCR products were separated onto 1.5% agarose gel and visualized by ethidium bromide staining. Real time-PCR was performed using ABI StepOnePlus real time PCR system with gene-specific primers and Smart Quant Green Master Mix with dUTP & ROX (Protech, PT-GL-SQGLR-V3). The mRNA of β-actin was used to standardize the total amount of cDNA on real-time PCR.

### Human LCN2 enzyme-linked immunosorbent assay (ELISA)

ELISA was performed using Human LCN2/NGAL DuoSet ELISA Kit (R&D Systems, DY1757) according to the manufacturer's instructions. The absorbance at 450 nm was measured using a microplate reader.

### Detection and quantification of acidic vesicular organelles with acridine orange

Autophagy is the process of packaging cytoplasmic proteins into the lytic component and characterized by the formation of acidic vesicular organelles (AVOs). After treatment for 48 h, cells were washed in PBS and stained with acridine orange (Sigma, A6014) (1 μg/ml) in serum-free LHC-9 medium for a period of 15 min, then washed twice with PBS and suspended in LHC-9. To observe the formation of AVOs, the cells were detected under a red filter fluorescence microscope and quantified using flow cytometry.

### HK activity assay

HK was assayed according to the manufacturer's instructions using HK Colorimetric Assay Kit (Biovision, K789-100). To assay total HK activity after treatment with NiCl_2_ and metformin, cell lysates were homogenized with ice cold HK Assay Buffer and the supernatant was collected with reaction mixture. This was followed by incubation for 5 min at room temperature and measurement at excitation wavelength of 450 nm for 30 min. Specific activity was determined using NADH standard. All experiments were repeated at least three times.

### VZV-G pseudotyped lentivirus-shRNA system

RNAi reagents were obtained from the National RNAi Core Facility located at the Institute of Molecular Biology/Genomic Research Center, Academia Sinica. Individual clones were identified by their unique TRC number: shGFP TRCN0000072178 (responding sequence: CAA CAG CCA CAA CGT CTA TAT) and shLuc TRCN0000072246 (responding sequence: CAA ATC ACA GAA TCG TCG TAT) for vector control; shHK2 (27) TRCN0000232927 (responding sequence: TGA CGA CAG CATC ATT GTT AA) and shHK2 (82) TRCN0000195582 (responding sequence: CCA AAG ACA TCT CAG ACA TTG) targeted to HK2; shLCN2 (89) TRCN0000060289 (responding sequence: CCA GCA TGC TAT GGT GTT CTT) and shLCN2 (90) TRCN0000060290 (responding sequence: GTA CTT CAA GAT CAC CCT CTA) targeted to LCN2; shLC3 (87) TRCN0000243387 (responding sequence: GGT GAT CAT CGA GCG CTA CAA) and shLC3 (91) TRCN0000243391 (responding sequence: AGC GAG TTG GTC AAG ATC ATC) targeted to LC3. The cells were selected with 2 μg/ml puromycin (Sigma, P8833).

### Detection of intracellular nickel

Intracellular mobilized ionic nickel was detected after NiCl_2_ and metformin treatment using green fluorescent Newport Green^TM^ DCF diacetate indicator dye (Invitrogen, N7991). The treated cells were washed twice with HBSS and incubated with 1 μM Newport Green^TM^ DCF diacetate in LHC-9 for 30 minutes. Then, they were washed twice with HBSS after recovery in LHC-9 containing Newport Green^TM^ DCF diacetate. Green fluorescence was visualized under an Olympus CK40 fluorescence microscope.

### pEGFP-LC3 plasmid transfection

pEGFP-LC3 expression vector was purchased from Addgene (#21073). The pEGFP-LC3 plasmid is a pEGFP-C1 plasmid inserted into microtubule-associated protein 1 light chain 3 (LC3) cDNA at the C-terminus and green fluorescent protein (GFP) at the N-terminus [69]. pEGFP-LC3 fusion protein was used to visualize the autophagosomes in cells. Transfection was performed on 24-well plates with coverslips, with 1 μg plasmid in each well and jetPEI transfection reagent (Polyplus-transfection, 101-10). This was followed by incubation overnight. The medium was removed and fresh medium containing NiCl_2_ and metformin was added to the wells for 48 h. After exposure, the cells were washed twice with PBS and fixed in 3.7% paraformaldehyde-PBS for 10 min at room temperature. Observation of GFP-LC3 puncta in cells was carried out under confocal microscope (ZEISS LSM510 META).

### Detection of intracellular reactive oxygen species (ROS)

Cellular ROS was detected using the fluorescence probe 2′, 7′-dichlorodihydrofluorescein diacetate (H_2_DCFDA) (Invitrogen, D399). After nickel and metformin treatment for 48 h, BEAS-2B cells were stained with 20 μM H_2_DCFDA at 37°C for 30 min in the dark. Then washed twice with PBS and harvested cell in PBS contained with 5% FBS. The fluorescence intensity was analyzed using flow cytometry.

### Expression analysis of the cancer genome atlas lung squamous cell carcinoma and lung adenocarcinoma data

Gene expression data were obtained from The Cancer Genome Atlas (TCGA) lung squamous cell carcinoma (LUSC) and lung adenocarcinoma (LUAD) datasets (https://tcga-data.nci.nih.gov/tcga). The datasets contain data from 463 LUSC samples with 50 adjacent normal tissue samples and 488 LUAD samples with 57 adjacent normal tissue samples, respectively.

### Immunohistochemistry

Antibodies against HK2 (2867) and LC3B (3868) were obtained from Cell Signaling Technology. Negative controls were used, leaving out the primary antibody. Immunohistochemical methods were carried out using conventional streptavidin peroxidase method according to the manufacturer's (Dako) LSAB Kit (K675) procedure. Slides were visualized using 3,3′-diamino-benzidine tetrahydrochloride as a substrate. The control slide (Dako, T1076) and semi-quantitative H scores of HK2 immunoreactivity were determined by multiplying the proportional scores of stained cells by their immunoreactivity intensity. All immunohistochemical staining cases were examined by two pathologists (Pei-Ru Wu and Kun-Tu Yeh, Department of Pathology, Changhua Christian Hospital, Changhua, Taiwan), and a final agreement was obtained for each score at a discussion microscope.

### Statistical analysis

Statistical analyses were performed using SPSS statistical software (version 18.0; SPSS, Inc., Chicago, IL). All statistical tests were two-sided, and a *p* value of less than 0.05 was considered statistically significant. Values presented are the means ± standard deviation (SD) of at least three independent experiments.

## SUPPLEMENTARY MATERIALS FIGURES AND TABLES



## Abbreviations

HK2: hexokinase 2
LCN2: lipocalin 2
NiCl_2_: nickel chloride
EMT: epithelial-mesenchymal transition
ROS: reactive oxygen species
KEGG: Kyoto Encyclopedia of Genes and Genomes
AVO: acidic vesicular organelles
2-DG: 2-deoxy-D-glucose
H_2_DCFDA: 2′, 7′ -dichlorodihydrofluorescein
NAC: N-acetyl-cysteine
p70S6K: p70 ribosomal protein S6 kinase
MFFs: mouse embryonic fibroblast
CQ: chloroquine
TCGA: The Cancer Genome Atlas
TLR4: Toll-like receptor 4
miRNAs: microRNAs
HIF: hypoxia-inducible factor
LUSC: lung squamous cell carcinoma
LUAD: lung adenocarcinoma
