# Supplementary material for: Metformin alleviates nickel-induced autophagy and apoptosis via inhibition of hexokinase-2, activating lipocalin-2, in human bronchial epithelial cells
Source: Oncotarget. 2017 Nov 6;8(62):105536–52. doi: 10.18632/oncotarget.22317 (PMC5739657; doi:10.18632/oncotarget.22317)
Supplement: Supplementary file 1 [file oncotarget-08-105536-s001.pdf]

Metformin alleviates nickel-induced autophagy and apoptosis via inhibition of hexokinase-2, activating lipocalin-2, in human bronchial epithelial cells

SUPPLEMENTARY MATERIALS

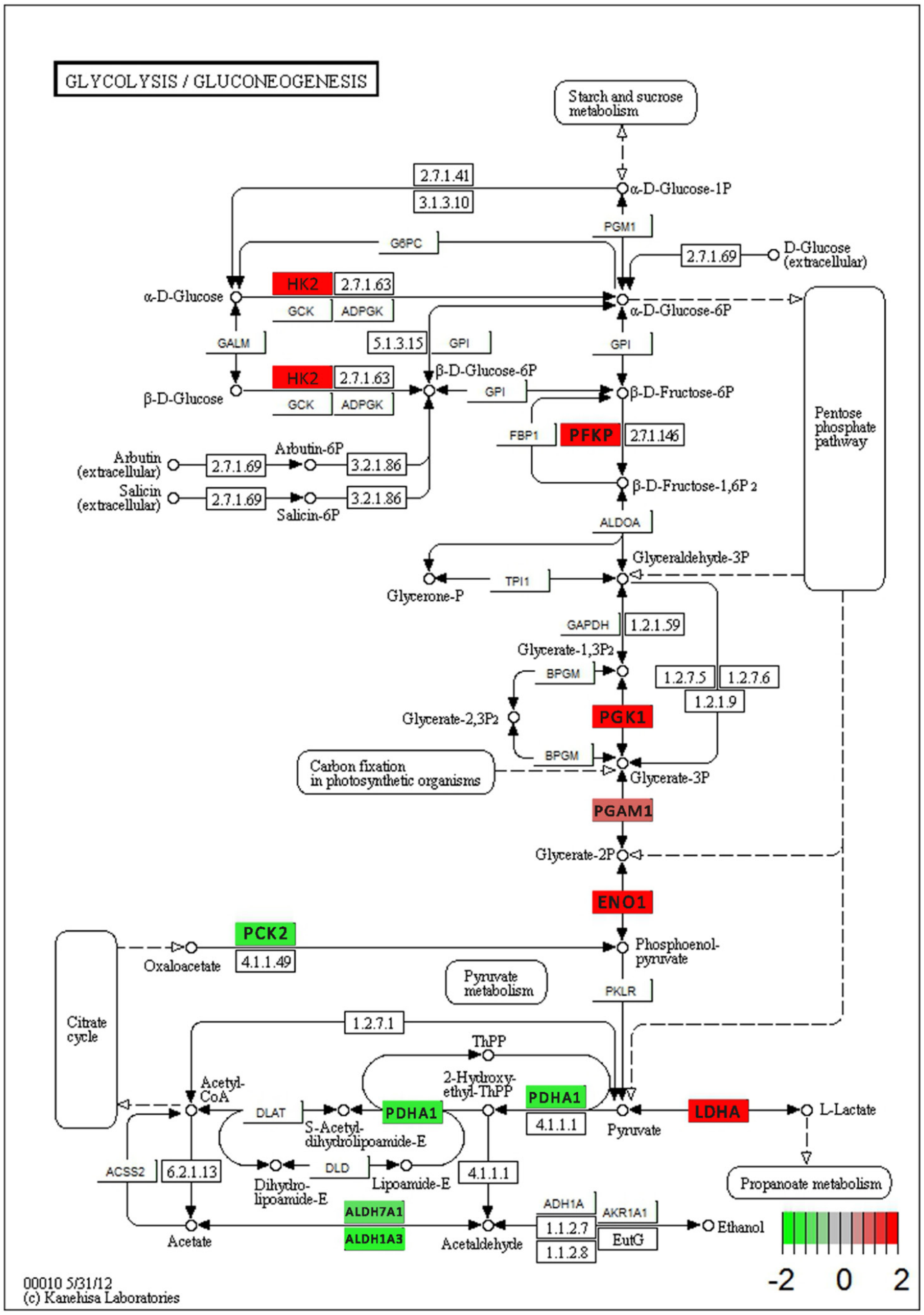

Supplementary Figure 1: Glycolysis/Gluconeogenesis pathway as defined in the KEGG Pathway database. Green and red boxes indicate down- and up-regulation, respectively.

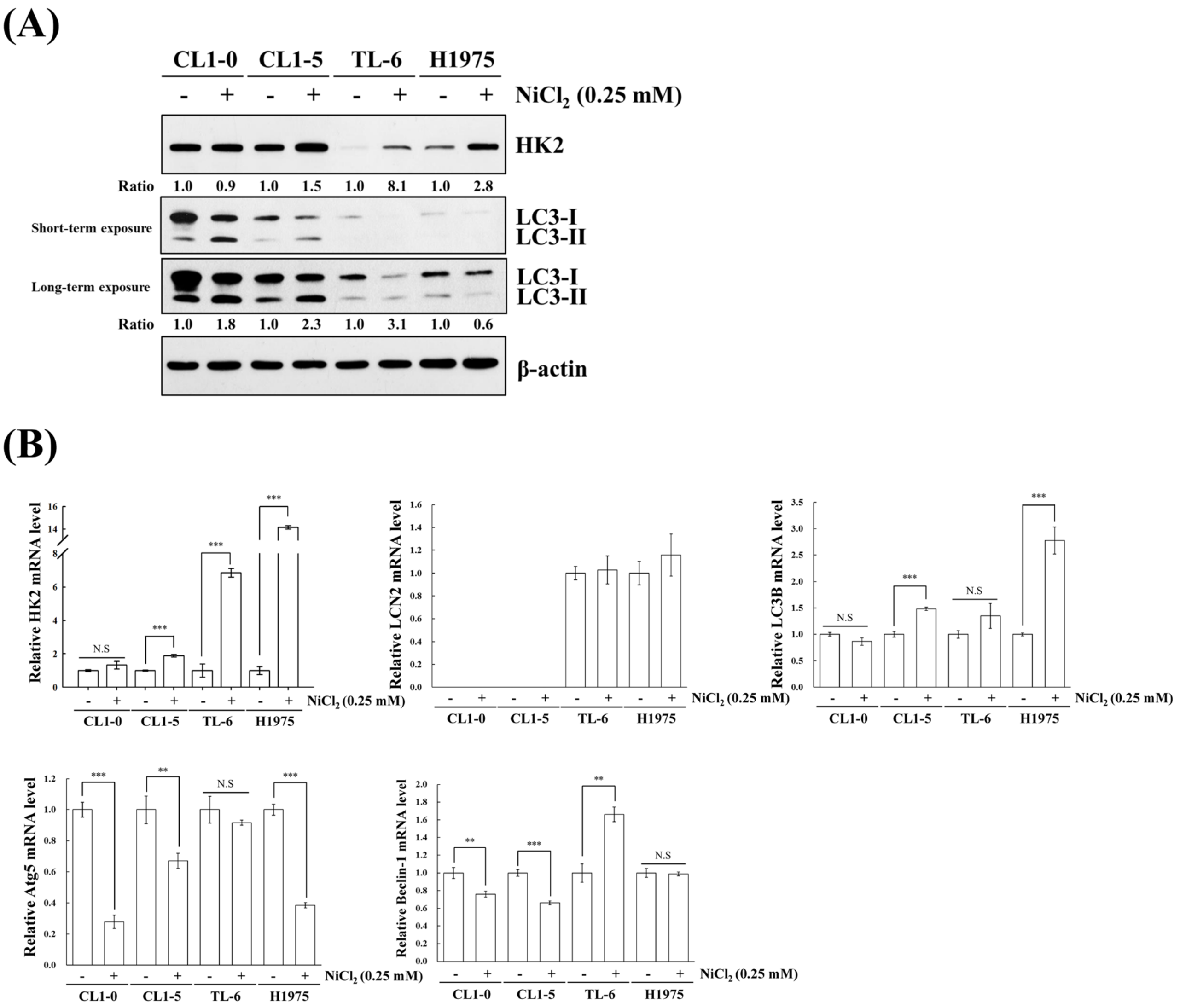

Supplementary Figure 2: The expressions of HK2 and LC3B are upregulated under $NiCl_2$ exposure in several lung cancer cell lines. CL1-0, CL1-5, TL-6 and H1975 cells (5×10^5 cells/ 6 cm dish) were treated with $NiCl_2$ (0, 0.25 mM) for 48 h. **(A)** The protein levels of HK2 and LC3B were determined on western blot. β-actin was used as internal control. **(B)** The mRNA levels of HK2, LCN2, LC3B, Atg5 and Beclin-1 were detected by real-time PCR. $^{**}p<0.01$ and $^{***}p<0.001$, two-tailed t test. N.S, no significance.

Supplementary Table 1: Glycolysis-related gene mRNA fold changes in $NiCl_2$-exposure BEAS-2B cells

Gene symbol	Processed signal (raw)		Fold change
	Control	$NiCl_2$ 0.5 mM	
Hexokinase 2 (HK2)	176.02	4212.95	38.50
Homo sapiens enolase 1 (ENO1)	10359.77	72121.87	6.96
Phosphofructokinase, platelet (PFKP)	8174.27	17985.48	2.20
Phosphoglycerate mutase 1 (PGAM1)	1460.91	1647.05	1.12
Phosphoglycerate kinase 1 (PGK1)	16115.57	48727.95	3.02
Phosphoenolpyruvate carboxykinase 2 (PCK2)	5354.25	511.41	0.10
Lactate dehydrogenase A (LDHA)	5713.74	22830.16	3.99
Pyruvate dehydrogenase alpha 1 (PDHA1)	4910.544	940.437	0.19

[*]Cell were exposed to 0.5 mM $NiCl_2$ for 48 h.

Supplementary Table 2: Relationships between HK2 H-score and clinical characteristics

Variable	Number	HK2 expression		p Value
		Non-detectable (%)	Detectable (%)	
Age (yrs)				
≦55	11	05 (45.5%)	06 (54.5%)	
> 55	61	43 (70.5%)	18 (19.5%)	0.105
Gender				
Female	36	24 (66.7%)	12 (33.3%)	
Male	36	24 (66.7%)	12 (33.3%)	1.000
Grade				
I	11	08 (72.7%)	03 (27.3%)	
II, III	61	40 (65.6%)	21 (34.4%)	0.643
Stage				
I, II	48	28 (58.3%)	20 (41.7%)	
III, IV	24	20 (83.3%)	04 (16.7%)	**00.034***
Tumor T status				
T1, 2	19	36 (61.0%)	23 (39.0%)	
T3, 4	40	12 (92.3%)	01 (07.7%)	**0.030***
Lymph node status				
N0	46	29 (63.0%)	17 (37.0%)	
N1, 2, 3	26	19 (73.1%)	07 (26.9%)	0.386
Metastasis				
M0	57	34 (59.6%)	23 (40.4%)	
M1	15	14 (93.3%)	01 (6.70%)	**00.014***
LC3B expression				
- (Absence)	55	35 (63.6%)	20 (36.4%)	
+ (Presence)	17	13 (76.5%)	04 (23.5%)	0.327
